# Persistence of Anti SARS-CoV-2 Antibodies in Breast Milk from Infected and Vaccinated Women after *In Vitro*-Simulated Gastrointestinal Digestion

**DOI:** 10.3390/nu14102117

**Published:** 2022-05-19

**Authors:** Joaquim Calvo-Lerma, Pierre Bueno-Llamoga, Christine Bäuerl, Erika Cortés-Macias, Marta Selma-Royo, Francisco Pérez-Cano, Carles Lerin, Cecilia Martínez-Costa, Maria Carmen Collado

**Affiliations:** 1 Institute of Agrochemistry and Food Technology—National Research Council (IATA-CSIC), 46980 Paterna, Valencia, Spain; pdbuella@edu.upv.es (P.B.-L.); christine.bauerl@iata.csic.es (C.B.); ercorma@iata.csic.es (E.C.-M.); mselma@iata.csic.es (M.S.-R.); 2 Section of Physiology, Department of Biochemistry and Physiology, Faculty of Pharmacy and Food Science, University of Barcelona (UB), 08028 Barcelona, Catalonia, Spain; franciscoperez@ub.edu; 3 Institute of Research in Nutrition and Food Safety (INSA), University of Barcelona (UB), 08921 Santa Coloma de Gramenet, Catalonia, Spain; 4 Endocrinology Department, Institut de Recerca Sant Joan de Déu, Hospital Sant Joan de Déu, 08950 Barcelona, Catalonia, Spain; carles.lerin@sjd.es; 5 Department of Pediatrics, Hospital Clínico Universitario, University of Valencia, 46010 Valencia, Valencia, Spain; cecilia.martinez@uv.es; 6 Nutrition Research Group of INCLIVA, 46010 Valencia, Valencia, Spain

**Keywords:** breastfeeding, anti-SARS-CoV-2, IgA, IgG, breast milk, *in vitro* digestion, colonic fermentation, gut microbiota, lactation

## Abstract

Breastfeeding is key for infant development and growth. Breast milk contains different bioactive compounds including antibodies. Recent studies have demonstrated the presence of breast milk SARS-CoV-2 antibodies after maternal infection and vaccination. However, the potential impact on the infant has not been explored yet. As a first step, we aimed at assessing the potential persistence of SARS-CoV-2 IgA and IgG antibodies from infected and vaccinated women in the gastrointestinal tract of the infants by means of an *in vitro*-simulated gastrointestinal digestion approach. Breast milk samples from 10 lactating women receiving mRNA vaccination against SARS-CoV-2 (n = 5 with BNT162b2 mRNA and n = 5 with mRNA-1273) and also, COVID-19 infected (n = 5) were included. A control group with women with no exposure to the virus (n = 10 pre-pandemic) were also studied. The presence of IgA and IgG SARS-CoV-2 antibody levels was determined by ELISA after the gastric and intestinal stages. The impact of digested antibodies on infant gut microbiota was tested by simulating colonic fermentation with two different fecal inoculums: infants from vaccinated and non-vaccinated mothers. Specific gut microbial groups were tested by targeted qPCR. *In vitro* infant gastrointestinal digestion significantly decreased the levels of both anti-SARS-CoV-2 IgA and IgG. However, both remained resistant in all the study groups except in that evaluating breast milk samples from infected women, in which IgG was degraded below the cut-off values in the intestinal phase. No effect of the antibodies on microbiota were identified after digestion. In conclusion, antibody levels against SARS-CoV-2 are reduced after *in vitro*-simulated gastrointestinal tract but remain present, so a positive biological effect could be expected from this infant immunization pathway.

## 1. Introduction

The COVID-19 pandemic has shifted research priorities in several disciplines and areas of knowledge, including the field of maternal–infant health. Since then, developing reliable and effective vaccines to revert transmission, understanding transmission vectors, implementing preventing strategies against infection and finding cures to palliate the symptoms have become main research concerns [1].

Vaccination with any of the available vaccines is a priority, as the current figure for full vaccination is higher than 80% of the adult population in Europe [2]. A specific group deserving attention in this sense are lactating women who have been in contact with the virus or virus-specific components, either via infection or vaccination, respectively. On the one hand, the absence of SARS-CoV-2 RNA in breast milk has been demonstrated and therefore the safety of breastfeeding has been confirmed [3]. On the other hand, the presence of specific SARS-CoV-2 antibodies has been reported in breast milk, constituting a pathway for antibody transmission that could exert a protective effect on the lactating infant [3]. In this sense, while most antibodies in human milk are IgA, the study of other Ig such as IgG and its isotypes are gaining attention in the last years [4] and in fact it is known that the vaccine induces milk IgG antibodies [5].

However, little is known about the persistence of these antibodies along the infant gastrointestinal digestion so their potential protective role remains unknown. Moreover, response to vaccination has shown to differ according to the type of the administrated vaccine, and variable effects -including the persistence of antibodies in plasma over time- have been described. In addition, these outcomes are different when comparing vaccinated or infected populations [6,7]. Altogether, current evidence encourages further studies regarding the levels and the persistence of anti-SARS-CoV-2 antibodies in breastmilk in infected and vaccinated women and their impact on infants’ health.

As a preliminary step, the present proof-of-concept study raises a reasonable hypothesis that differences in the presence and resistance during digestion of anti-SARS-CoV-2 antibodies in breast milk could be assessed depending on the type of vaccine or the fact of being infected. In addition, antibodies in breast milk during gastrointestinal digestion could be degraded because of the effect of the pH or the enzymatic action, as previously reported in other contexts [8,9]. Additionally, antibodies in breast milk have been reported to modulate infants’ gut microbiota [10], so that changes in gut microbiota could be also a possible effect in the case of the SARS-CoV-2 antibodies.

Therefore, the aim of the present exploratory study was to assess the persistence of breast milk anti SARS-CoV-2 IgA and IgG along in vitro-simulated gastrointestinal digestion, and the influence of their origin, depending on the type of the maternal contact with the virus, either infection or vaccination. Secondarily, the study aimed at evaluating the potential impact of these breast milk antibodies on the infant microbiota by in vitro colonic fermentation.

## 2. Materials and Methods

### 2.1. Subjects and Study Design

From a prospective cross-sectional and observational study in Spain, a pilot study with breast milk samples from 10 lactating women receiving mRNA vaccination against SARS-CoV-2 in 2021 was carried out (n = 5 with BNT162b2 mRNA, BioNTech/Pfizer and n = 5 with mRNA-1273, Moderna) (NCT04751734). The vaccinated participants, within the vaccination priority groups (frontline health care workers) established by the Spanish Government, had received two doses of mRNA vaccines. A group with confirmed SARS-CoV-2 infection by PCR (n = 5) was also included (NCT04768244). In addition, breast milk samples from 10 lactating women from the pre-pandemic era were selected (NCT03552939) as controls to set cut-off values for specific anti-SARS-CoV-2 IgA and IgG. The participants received information and written consent was obtained before enrolment. All procedures were in accordance with the ethical standards approved by the Ethical Committee of the Hospital Clínico Universitario (No. 2020/133) and CSIC (No. 061/2021). The infants’ fecal samples for the simulation of colonic fermentation were also obtained under the same ethical approvals.

**Figure 1:**
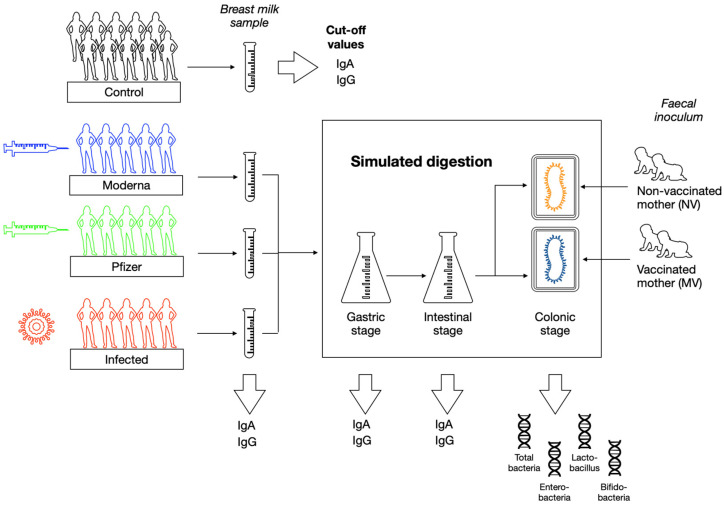
Overview of the study design.

On the collected breast milk samples from the different groups of lactating women, anti-SARS-CoV-2 IgA and IgG were analyzed prior and after simulation of infant gastrointestinal digestion. The obtained digesta was then subjected to colonic fermentation to assess the possible impact of the type of contact with the virus on the colonic microbiota. The study design overview is provided in Figure 1.

### 2.2. Breast Milk Samples Collection and Processing

The breast milk samples were collected after 2 weeks of the second dose, as this point corresponded with the maximum detectable levels of anti-SARS-CoV-2 IgG and IgA, as described in a previous study [3]. Samples from SARS-CoV-2 infected mothers were collected at a median of 6 days (1.5, 146, 1st and 3rd quartile) post-diagnosis. In addition, the samples from the control group (n = 10) were obtained before the pandemic to establish *cut-off* values. Breast milk was collected mainly by the use of a sterile pumper in sterile bottles to normalize the collection among participants and morning collection was recommendable. Samples were stored immediately at −20 °C and later stored at −80 °C as detailed elsewhere [11]. For the analytical determinations, the samples were thawed, and an aliquot of 4 mL was directly used for simulation of digestion. The rest of the sample was centrifuged (14,000 rpm, 4 °C, 10 min) to remove fat and the resulting skimmed fraction was used for antibodies determination.

### 2.3. Quantification of Specific SARS-CoV-2-IgA and IgG Concentration in Breast Milk

Anti-SARS-CoV-2 IgA and IgG were analyzed in the undigested breast milk samples and after the gastric and intestinal stages of simulated *in vitro* digestion to determine the possible degradation by the action of pH and digestive enzymes. Levels of receptor-binding domain (RBD)-specific antibodies were analyzed as described by Bäuerl et al. (2021) [3] using a previously published ELISA protocol [12,13]. RBD protein was produced under HHSN272201400008C and obtained through BEI Resources, NIAID, NIH: Spike Glycoprotein RBD from SARS-CoV-2, Wuhan-Hu-1 with C-Terminal Histidine Tag, Recombinant from HEK293T Cells, NR-52946. Briefly, 96-well immunoplates (Costar) were coated with 2 µg/mL of RBD overnight at 4 °C, blocked in 3% (*w*/*v*) milk powder in PBS containing 0.1% Tween 20. Samples were incubated in a 1:4 dilution for 2 h and after 3 washing steps incubated with anti-human IgA (α-chain-specific) horseradish peroxidase (HRP) antibody (Thermo-Fisher Scientific; A18781; 1:6.000) and anti-human IgG (Fc-specific) HRP antibody (Sigma-Aldrich St Louis, MO, USA; A0170; 1:4.000) for 1 h. The plates were developed with 3,3′,5,5′-Tetramethylbenzidine and reactions were stopped with 50 μL of 2 M sulfuric acid. Absorbance at 450 nm was read in a ClarioStar (BMG Labtech, Ortenberg, Germany) microplate reader. 

Standard curves were prepared using previously analyzed human milk samples with the highest optical density (OD) values for both IgA and IgG [3]. For IgA, three samples from infected mothers, and for IgG, ten samples from vaccinated mothers, were pooled and were assigned a value of 3000 arbitrary units (AU). The standard curves were plotted using 4-parameter non-linear regression function in GraphPad Prism 8.4.3. Pre-pandemic samples were used to determine positive cut-off values for each antibody isotype, defined as mean + two standard deviations.

### 2.4. In Vitro Infant Digestion

Whole breast milk samples (800 µL) were subjected to static *in vitro* digestion according to a protocol specifically adapted for lactating infants [14]. The gastric stage was reproduced by the addition of the simulated gastric fluid containing pepsin (268 U/mL) and gastric lipase (19 U/mL) to the milk sample in proportion 6:4 (*v*/*v*) in a 10 mL tube, and adjusting the pH with HCl (1 M) to 5.3. Tubes were then placed in a thermostated chamber at 37 °C in agitation at 55 rpm, and the gastric stage was allowed for 60 min. Then, the intestinal stage was started by adding in proportion 4:6 (*v*/*v*) the simulated intestinal fluid containing pancreatin (90 lipase U/mL) and bile salts (3.1 mM). After adjusting the pH to 6.6 with NaOH (1 M), another 60 min were allowed for the intestinal stage in the agitated chamber. At the end of the simulation, the samples were immediately placed in ice to stop any enzymatic reaction. At the end of the gastric and the intestinal stages, 0.2 mL aliquots were taken to quantify IgG and IgA levels. Aliquots were kept frozen at −80 °C until further analysis.

### 2.5. In Vitro Colonic Fermentation

The resulting digesta from simulated gastrointestinal digestion were used for the simulation of colonic fermentation. A static method was selected, consisting of 2 mL fermentation glass vials with culture medium, fecal inoculum and the digested breast milk samples. 

For the fecal inoculum, fresh fecal samples of lactating infants were used. Two of the samples belonged to lactating infants whose mothers had received the two doses of the Pfizer vaccine (V) while the other two belonged to infants from non-vaccinated or infected mothers (NV) (Supplementary Table S1). Following this approach, we assessed possible changes in the baseline microbiota according to the maternal vaccination status. Samples were collected and transported in ice to the laboratory where bacteria were first isolated. Two pools of fecal samples (V and NV) were made by mixing the samples together. From the mixture, 3 g were obtained and mixed with NaCl (0.9%) in the proportion 1:10 (*w*/*v*) and head-over-heels agitated during 30 min. Then, the supernatant was collected and kept for further use as inoculum. The culture medium was prepared as previously described [15]. 

An initial stabilization phase was first established. To the fermentation vials containing 1250 μL of culture medium, 100 μL of the obtained inoculum were added and grown at 37 °C under anaerobic conditions for 24 h using an anaerobic atmosphere generation system (Anaerogen, Oxid). After the stabilization time, the digested samples (500 μL) were injected in anaerobic conditions to initiate colonic fermentation. At 24 and 48 h from this moment, aliquots were taken for further microbiota analyses. Control vials, including culture medium and inoculum, but not samples, were also prepared and aliquoted at the same time points.

### 2.6. Microbial Profile by Quantitative PCR Analysis (qPCR)

Total DNA was extracted from the colonic digesta after *in vitro* fermentation (approx. 100–200 mg) using the Master-Pure DNA extraction Kit (Epicentre, Madison, WI, USA) following the manufacturer’s instructions with the following modifications: samples were treated with lysozyme (20 mg/mL) and mutanolysin (5 U/mL) for 60 min at 37 °C and a preliminary step of cell disruption with 3-μm diameter glass beads followed by 1 min at 2000 oscillations by bead beater. Purification of the DNA was performed using DNA Purification Kit (Macherey-Nagel, Duren, Germany) according to manufacturer’s instructions. DNA concentration was measured using Qubit^®^ 2.0 Fluorometer (Life Technology, Carlsbad, CA, USA) [3].

qPCR was targeted to quantify total bacteria, Enterobacteriaceae family, Bifidobacterium and Lactobacillus genus, as previously described (Collado et al., 2008; Mira-Pascual et al., 2015). The qPCR amplification and detection were performed in duplicate on a LightCycler 480 Real-Time PCR System (Roche Technologies, Mannheim, Germany). Each reaction mixture of 10 μL was composed of SYBR Green PCR Master Mix (Roche), 0.5 μL of each primer (concentration 10 mmol/L), and 1 μL of DNA template. The bacterial concentration in each sample was calculated by comparison with the Ct values obtained from standard curves that were obtained by serial 10-fold dilutions of specific DNA fragments.

Data were expressed as total number of copies per mL of sample at baseline and after 48 h of colonic fermentation and also as the logarithm. Then, changes after 48 h of colonic fermentation with respect to baseline values were expressed as the change percentage (0–100).

### 2.7. Statistical Analyses

Data were summarized as mean and standard deviation or median and 1st and 3rd quartiles (Q). For descriptive graphical representation of Ig results after simulated digestion, Graphpad software was used. Statgraphics Centurion-XV was used to explore potential differences in Ig values between groups of samples (vaccinated with Moderna or Pfizer and infected women) and between the different timepoints through gastrointestinal simulation, by means of Kruskal–Wallis tests, with a confidence interval of 95% (*p* < 0.05). The same analysis was applied to determine statistically significant differences between microbiota in the V and the NV inoculums, and after 48 h of colonic fermentation.

## 3. Results

### 3.1. Subjects Characteristics

Characteristics of the volunteer vaccinated, infected or control mothers who donated the breast milk samples are compiled in Table 1. Notably, the samples from the infected group were collected at an earlier timepoint than the rest of the groups.

### 3.2. Persistence of Antibodies along In Vitro Gastrointestinal Digestion

**Figure 2:**
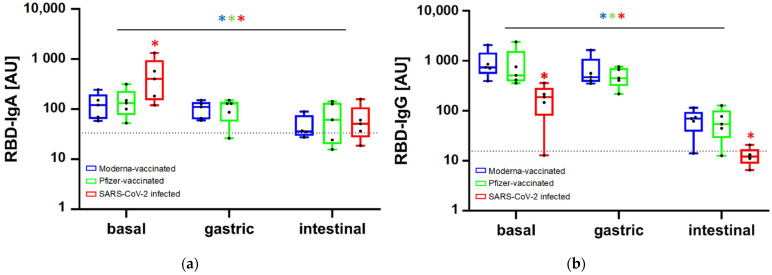
Change in anti-SARS-CoV-2 IgA (**a**) and IgG (**b**) levels along the simulated gastrointestinal digestion in the breast milk samples from the three study groups. In the SARS-CoV-2 infected group an aliquot at the gastric stage was not collected due to the scarce amount of sample. The horizontal dotted line indicates the cut-off value established as per pre-pandemic breast milk samples. * indicates statistically significant differences (*p* < 0.05) between groups of samples within each timepoint and between time points considering each group of samples (blue * for Moderna-vaccinated, green * for Pfizer vaccinated and red * for SARS-CoV-2 infected).

Basal anti-SARS-CoV-2 IgA values in breast milk samples prior simulated digestion were 132 (76, 232) AU (median (1st and 3rd quartile) and 121 (64, 197) AU in the Pfizer and Moderna vaccinated groups, respectively, with no statistically significant differences. However, when comparing these results with the infected group, IgA values were significantly higher, with a median value of 401 (151, 951) AU (Figure 2a). In contrast, focusing on IgG, samples from the infected group showed significantly lower basal levels [188 (79, 287)] AU than samples from the vaccinated groups [511 (386, 1577) AU in Pfizer and 740.9 (549, 1480) AU in Moderna] (Figure 2b).

Different persistence patterns were found after *in vitro* digestion, although a common observation was that both anti-SARS-CoV-2 IgA and IgG levels decreased, especially after the simulation of the intestinal stage. Gastric simulation only accounted for minor non-significant changes (Figure 2). In the infected group the aliquot at the gastric stage was not collected due to the scarce amount of sample. Despite IgA levels were initially higher in the infected group, values after simulated digestion decreased to the same magnitude than the vaccinated groups, with no significant differences among them. Concretely, after intestinal digestion the IgA values were 61 (29, 134) AU in the Pfizer group, 41 (27, 109) AU in the infected and 36 (29, 76) AU in Moderna. However, the median values in the three groups remained above the cut-off values established as per the pre-pandemics control group. Moving onto the change of anti-SARS-CoV-2 IgG throughout simulated digestion, the final values were significantly lower and kept the same relative proportion between groups as the initial values in the breast milk samples prior digestion. So, the significantly lower IgG levels in the vaccinated group were kept lower compared to the vaccinated groups after simulation of the intestinal stage. At this final point, anti-SARS-CoV-2 IgG levels were 70 (38, 95) AU and 54 (29, 102) AU in samples from vaccinated mothers with Moderna and Pfizer, respectively, and 12 (9, 17) AU in those from infected mothers. In addition, the range of anti-SARS-CoV-2 IgG levels in samples from the infected group included values below the cut-off, suggesting the lack of persistence of this immunoglobulin.

### 3.3. Effect of Vaccination or Infection on the Colonic Microbiota Profile

Following gastrointestinal simulation, the digested samples were subjected to *in vitro* colonic fermentation to complete the digestion process and to ascertain the potential impact of the presence of antibodies on the colonic microbiota profile. 

Two different types of inoculums were used, belonging to fecal samples from infants whose mothers had received either the Pfizer vaccination (V) or had not been vaccinated or infected (NV) in order to consider the previous exposition to the vaccines during lactation and the potential impact of the vaccine in breast milk and the infant’s gut. Baseline composition of the inoculums, however, presented no significant differences between each other. Expressed as the logarithm of the total number of copies of for each taxa gene/mL of colonic digesta, mean values were, in the V and NV inoculum, respectively, the following: 7.61 and 7.47 (*p* > 0.05) for total bacteria, 6.28 and 5.91 (*p* > 0.05) for *Enterobacteriaceae* family members, 4.07 and 4.12 (*p* > 0.05) for *Lactobacillus*, and 5.13 and 4.98 (*p* > 0.05) for *Bifidobacterium* genus.

**Figure 3:**
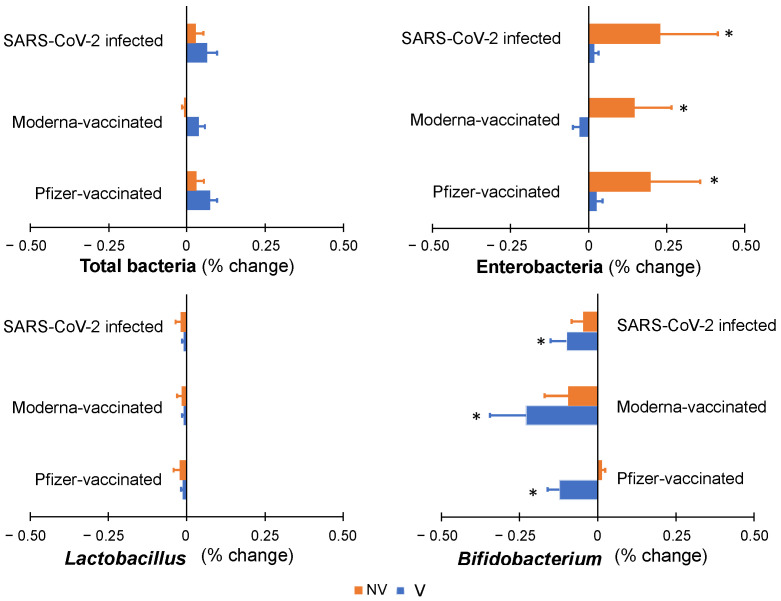
Colonic microbiota profile along simulated colonic fermentation of infants from vaccinated (V) and non-vaccinated or infected (NV) mothers, using as substrates digested breast milk samples in previous gastrointestinal simulation, containing anti-SARS-CoV-2 antibodies as a result of maternal vaccination (with Pfizer or Moderna) or infection. * indicates statistically significant differences with respect to baseline values prior to colonic fermentation.

Minor changes were detected after 48 h of simulation of colonic fermentation, being smaller than 1% in total bacteria and in the three genera, either by increasing or decreasing the bacterial abundances (Figure 3). Regarding total bacteria and *Lactobacillus*, no significant changes were detected compared to baseline composition or between the NV and MV inoculums. Although only modest differences were observed, the largest changes were found in Enterobacteria, which increased up to 0.25% with respect to baseline inoculum in the digesta of the samples from all three groups (Pfizer, Moderna and Vaccinated) in the case of the NV inoculum. This significant change was not observed with the V inoculum. On the other hand, the *Bifidobacterium* genera decreased up to −0.23% in the samples from all the groups but only with the V inoculum. Overall, the presence of anti-SARS-CoV-2 antibodies in breast milk samples had a non-significant effect on infants’ colonic microbiota.

Complementarily to the characterization of microbial genera, the metabolic activity was determined by means of measuring SCFA production (Supplementary Table S2). Only a significant difference was observed in the production of acetic acid between the samples of infected mothers when comparing the simulated colonic fermentation between the V and the NV inoculums.

## 4. Discussion

This study assessed the persistence of anti-SARS-CoV-2 antibodies in breast milk samples from vaccinated and infected lactating women in the infants’ digestive tract, by means of a simulated digestion approach. Our results showed that samples from the infected mothers had higher anti-SARS-CoV-2 IgA and lower IgG baseline values compared to the samples from vaccinated mothers. After gastric- and intestinal-simulated digestion, both anti-SARS-CoV-2 IgA and IgG values significantly decreased, but remained above control cut-off values in most of the cases. Later on, digested samples were subjected to *in vitro* colonic fermentation to assess the potential effect on the microbiota composition due to vaccination or infection with the virus, but non-significant results were found.

The main outcome of this exploratory research is that IgA and IgG anti-SARS-CoV-2 antibodies were not completely degraded after gastrointestinal digestion, except for breast milk IgG from infected mothers, which were the most affected by digestion. These results suggest the potential immunological activity of these Ig in the babies’ lumen during breastfeeding. In the study conducted by Pieri et al. (2021), one of the few related to specific anti-SARS-CoV-2 antibodies persistence after *in vitro*-simulated digestion, Pfizer-induced IgA levels were reported to be remarkably stable in the gastric phase but were degraded in the intestinal phase [16]. The difference with our study regarding Ig persistence in intestinal digestion may be attributed to the simulated conditions, as in our study these were adapted to better approach the gastrointestinal scenario in lactating infants [14], including a higher pH, different enzymatic concentrations and less duration. It is also worthy to point out that Pieri’s study was conducted with one sample and it included the Pfizer vaccine only, while ours used five breast milk samples per study group. 

Besides, anti-SARS-CoV-2 IgG levels after the *in vitro* digestion showed significant differences between samples from vaccinated and infected mothers, with the latter group below the *cut-off* values. These results suggest vaccination of breastfeeding mothers is an interesting tool to provide passive defenses against SARS-CoV-2, even more resistant than those generated in the context of infection. However, this difference could be related to the different lactating times when samples from the vaccinated and the infected groups were collected. In addition, the different Ig pattern induced in both contexts can be also a factor involved, as the virus-specific antibody profile in breast milk after intramuscular vaccination is characterized by an IgG-dominant response with low levels of IgA [17], whereas infection is related to a dominant early antibody response dominated by IgA [18]. 

Nevertheless, in other contexts, there are also reports of decreasing total IgA and total IgG in the gastric digestion, although IgG experimented a higher fall [19]. There is another study that evaluated the effect of gastric conditions on IgG levels without the effect of the milk matrix and it was observed that up to 25% of the original administered amount resisted the gastric conditions [20]. Most of IgA present in human milk is secretory IgA (sIgA) which is known as the primary protective agent of human milk [4], protecting against all types of pathogens, including viruses, by preventing prospective adherence onto the epithelial surfaces of the digestive system [21]. IgG also has protective effects in the gastrointestinal tract by neutralizing viruses and preventing attachment to the mucosa, although IgG is not as efficient as IgA in such function [19]. Contrarily to sIgA, IgG lacks the highly glycosylated secretory component and J chain, which is complexed to the dimeric form of IgA, and makes IgG more susceptible to proteolysis. This fact could explain the observed higher decrease in IgG after intestinal-simulated digestion. In our study we did not differentiate whether the presence of anti-SARS-CoV-2 IgA was secretory or not, but this is certainly crucial for future research, particularly considering the findings of a preliminary study in human milk that describes a lack of virus-specific sIgA after vaccination [17].

Overall, despite the reported persistence of IgA and IgG antibodies through digestion, it is not necessarily certain that both resistant Ig have the potential to neutralize the virus. *In vitro* neutralization assays using human milk from both infected and vaccinated mothers showed good virus-neutralizing capacity [22,23], but so far, no data exist for digested breast milk. In infants, other agents or mechanisms from the innate immune response-specific —apart from Ig—agents from the adaptive immune response could have protective effects against the virus, including lactadherins, lactoferrin, lysozyme or mucins [24,25,26]. 

Moving onto the effect of the presence of anti-SARS-CoV-2 IgA and IgG in breast milk on colonic fermentation, no changes related to total bacteria and the specifically assessed genera were observed in both types of inoculums and in any of the origins of breast milk. This finding suggests the safety of maternal vaccination regarding their impact on the infant’s colonic microbiota. Previous studies assessing the safety of other vaccines, such as rotavirus, have reported similar findings regarding the non-relevant effect on the colonic microbiota [27]. Although the secreted IgA in the gut is known to shape the composition of the intestinal microbiome, the underpinning mechanisms are still unknown [28]. In addition, the effect of digestion in the oral cavity on Ig degradation has not been assessed in the present study, and it also deserves consideration, as well as the impact these antibodies may have on the oral microbiome. Complementarily, the non-significant differences in the production of SCFA between the vaccinated and non-vaccinated inoculums and the samples from the different subjects’ group support the lack of changes in colonic microbiota after *in vitro* colonic fermentation. 

This study entails a limitation related to the *in vitro* methodology to study the persistence of antibodies through the gastrointestinal tract. However, digestion models are widely used for different purposes related to the follow-up of compounds in any of the digestion stages. Otherwise, in vivo approaches would not enable the access to the sampling sites (stomach, small and large intestine), or with considerable ethical concerns and burden for the subjects. In addition, the *in vitro* digestion model we used did not allow to reproduce intestinal absorption of the persistent antibodies. Moreover, the number of samples considered in each group is limited to five, but these samples are of unique value as collected at the appropriate and relevant timepoints of the pandemic. Another limitation relates to the reported decrease in the Ig levels after gastrointestinal digestion, while their most effective targeted effect could be in the oral cavity, which could not be assessed in the present study. So, future studies should assess the persistence of the anti-SARS-CoV-2 IgA and IgG in the intestinal content, simulating also the action of the oral enzymatic and microbial metabolism; and explore the action of possible Ig-degrading proteases from the microorganisms that could be present in the infant gut microbiota [29,30,31,32].

## 5. Conclusions

In conclusion, the present exploratory pilot study suggests that, although the levels are decreased, there is persistence of breast milk anti-SARS-CoV-2 antibodies during gastrointestinal digestion with no implications on the baby’s colonic microbiota composition and activity. Despite being limited to the *in vitro* framework and the reduced sample size, this study suggests the possible role of breast milk anti-SARS-CoV-2 antibodies in babies from vaccinated and infected mothers. These results should be further validated in humans and should explore their real preventive effect.

## Figures and Tables

**Table 1:** Clinical and demographic characteristics at the moment of breast milk sample collection.

	Pfizer (n = 5)	Moderna (n = 5)	SARS-CoV-2 Infected (n = 5)	Controls (n = 10)
Maternal age (years, *median, 1st, 3rd Q*)	31 (29, 32)	35 (34.2, 37.2)	34 (31, 25)	37 (34, 38)
Lactation time (months, *median, 1st, 3rd Q*)	8 (7, 10)	8.5 (6.5, 11)	0.8 (0.5, 1.1)	6 (3.5, 60.5)
Maternal use of antibiotics	0/5	0/5	0/5	1/10
Type of delivery	Vaginal 2/5 C-Section 3/5	Vaginal 4/5 C-Section 1/5	Vaginal 5/5	Vaginal 8/10 C-Section 2/10
Exclusive breastfeeding	4/5	4/5	5/5	7/10

## Supplementary Materials

The following supporting information can be downloaded at: https://www.mdpi.com/article/10.3390/nu14102117/s1; Table S1: Characteristics of infant fecal sample donors; Table S2: Production of acetic acid (mM) after 48 h of simulated colonic fermentation of breast milk samples from vaccinated mothers with Pfizer and Moderna and infected mothers, using the inoculum of lactating infants from vaccinated (V) and non-vaccinated (NV) mothers.
