# Supplementary material for: Persistence of Anti SARS-CoV-2 Antibodies in Breast Milk from Infected and Vaccinated Women after In Vitro-Simulated Gastrointestinal Digestion
Source: Nutrients. 2022 May 19;14(10):2117. doi: 10.3390/nu14102117 (PMC9147794; doi:10.3390/nu14102117)
Supplement: Supplementary file 1 [file nutrients-14-02117-s001.zip › nutrients-1667970-supplementary.pdf]

Supplementary Materials

Persistence of Anti SARS-CoV-2 Antibodies in Breast Milk from Infected and Vaccinated Women after *In Vitro*-Simulated Gastrointestinal Digestion

Joaquim Calvo-Lerma [1,*], Pierre Bueno-Llamoga [1], Christine Bäuerl [1], Erika Cortés-Macias [1], Marta Selma-Royo [1], Francisco Pérez-Cano [2,3], Carles Lerin [4], Cecilia Martínez-Costa [5,6] and Maria Carmen Collado [1,*]

Table S1. Characteristics of infant faecal sample donors.

Sample Number	Pool Group	Donor Age (months)	Gender	Feed	Maternal Age (years)	Vaccination type	Vaccination time
1	V	5.5	Female	Mixed feeding	35	Pfizer	2nd dose in March 2021
2	V	4.5	Female	Breast milk	36	Pfizer	2nd dose in June 2021
3	NV	8	Female	Formula and complementary food	35	-	-
4	NV	4	Male	Mixed feeding	32	-	-

V, vaccinated mother; NV, non-vaccinated.

Table S2. Production of acetic acid (mM) after 48 h of simulated colonic fermentation of breast milk samples from vaccinated mothers with Pfizer and Moderna and infected mothers, using the inoculum of lactating infants from vaccinated (V) and non-vaccinated (NV) mothers.

	V inoculum	NV inoculum
Pfizer	$13.52 \pm 3.01^{a,A}$	$11.06 \pm 0.75^{a,A}$
Moderna	$11.47 \pm 1.32^{a,A}$	$10.58 \pm 0.74^{a,A}$
Infected	$15.97 \pm 3.57^{a,B}$	$9.27 \pm 1.43^{a,A}$

Different upper-case capital letters (A–B) mean significant differences ($p < 0.05$) between samples from the same subjects' group and different inoculums. Different lower-case letters (a, b) mean significant differences ($p < 0.05$) between the inoculum in the same subjects' groups.
